# Predictive Analysis of Factors Influencing Depression Status of Nurses in the COVID-19 Pandemic Intensive Care Unit

**DOI:** 10.3389/fpsyt.2021.596428

**Published:** 2021-11-16

**Authors:** Jing Li, Yanhua Zhang, Li Li, Wei Yi, Yiwei Hao, Yongjuan Bi

**Affiliations:** ^1^Department of Obstetrics, Beijing Ditan Hospital Capital Medical University, Beijing, China; ^2^Department of Intensive Care Unit, Beijing Ditan Hospital, Capital Medical University, Beijing, China; ^3^Department of Medical Records Room, Beijing Ditan Hospital, Capital Medical University, Beijing, China

**Keywords:** NCP, ICU nurses, depression, influencing factors, psychological interventions

## Abstract

**Purpose:** Understand the effects of the COVID-19 pandemic on depression in intensive care unit (ICU) nurses, analyze high-risk factors, and propose appropriate measures to maintain physical and mental health.

**Methods:** A total of 78 nurses in ICU of Beijing Ditan Hospital affiliated with Capital Medical University (Beijing area, COVID-19 patient designated hospital) were investigated with self-rating depression scale (SDS). The Cronbach'sαcoefficient was 0.874, the content validity was 0.853, and the internal consistency was good. General information for the questionnaire: gender, marriage, education, age, title, length of service, ICU years of service, COVID-19 pandemic training, concerns about the COVID-19 pandemic, and current health status.

**Results:** According to the SDS scale score, ICU nurses had a total depression score of 51.36 ± 11.667, and the prevalence rate of depression was 44.9% (35/78). Multi-line regression analysis shows that stress perception, work experience in critical diseases, education and other total scores are risk factors for the occurrence of depression.

**Conclusion:** Work experience in critical illness (β = 9.930, *P* < 0.001) had a positive predictive effect on the total score of depression, while stress perception (β = −0.884, *P* < 0.001) and education (β = −6.061, *P* < 0.001) had a negative predictive effect on the total score of depression, and explained 52.7% variation. These findings point to the need for interventions to address psychological distress and provide the necessary support.

## Introduction

Currently, COVID-19 has been alleviated in China, but the global epidemic is on the rise (1). The epidemic is characterized by unpredictability, sudden onset, rapid spread, complex causes, difficult treatment and severe disability. The number of infected patients has increased dramatically. In addition, there have been reports of COVID-19 pandemic nosocomial infections in China and other countries (2), which have a significant impact on health and physical and mental well-being of health care workers and can lead to depression. The intensive care unit (ICU) is the primary place for treating patients with severe COVID-19 and plays a key role in the fight against the COVID-19 pandemic. The World Health Organization (WHO) focuses on maintaining the physical and mental health of those involved in the relief effort and improving the physical and mental health of all employees (3). However, at present, there are few investigations on ICU nurses' depression, and there is a lack of operational psychological intervention methods. Usman et al. (4) discussions, that can be beneficial to reduce the psychological sufferings by ensuring the protection of the health-care workers to facilitate proper services in combating with the COVID-19 crisis. In addition, Sakib et al. (5) suggesting the urgent need to promote mental well-being in medical professionals. Therefore, it is urgent to identify the influencing factors of ICU nurses' depression. This study predicted and analyzed the influencing factors of depression state of nursing staff in designated hospitals for COVID-19 pandemic, and developed corresponding nursing measures, so as to provide reference for psychological intervention of ICU nurses during COVID-19 pandemic.

## Study Procedure and Participants

### Study Participants Inclusion Criteria

A questionnaire survey was conducted on 78 ICU nurses in Beijing COVID-19 pandemic designated hospital in March 2020 using cluster sampling. The inclusion criteria were as follows: (i) work in ICU; (ii) on duty; (iii) willing to participate in the survey. The exclusion criteria were as follows: (i) within 1 year of pregnancy and postpartum; (ii) family changes within 6 months of investigation; (iii) nurses with severe acute and chronic diseases; (iv) nurses with original mental and other mental illnesses. The studies involving human participants were reviewed and approved by the ethics committee of Beijing Ditan Hospital Capital Medical University Written informed consent from the participants was not required to participate in this study in accordance with the national legislation and the institutional requirements.

### Instruments

#### Study Procedure

A cross-sectional survey was conducted and the data were collected by “Questionnaire Star.” Each section is set as a mandatory option. The respondents filled in and submitted the questionnaire independently within 5 min. Participants completed the scale by scanning the code, input the ICU work information, and obtained SDS and Perceived stress scale (PPS).

#### Survey Tools: The Questionnaires Were Used as Research Tools

The specific contents of the questionnaire include:

(1) General information: Gender, marriage, education, age, job title, working years, ICU working years, COVID-19 pandemic training, concerns about COVID-19 pandemic, and current health status, work experience of critically ill patients (At least 1 years working experience in ICU), perceived stress scale (PPS), Confident to complete the work (independent willingness), etc.

(2) Self-Rating Depression Scale (SDS) (6): The questionnaire was compiled by William w. k. Zung in 1971. It is easy to use and can directly reflect the subjective feelings of patients with depression. There are 20 items in the scale, the positive score is 1, 2, 3, 4, and the reverse score is 4, 3, 2, 1. Reverse scoring question number: 2, 5, 6, 11, 12, 14, 16, 17, 18, and 20. The cumulative score of each item is the total rough score, which is multiplied by 1.25 to get the standard total score. A standard total score of <53 was not considered to be depressed, and a standard total score of more than 53 was considered to be depressed (53–63 was considered mild depression, 64–74 was considered moderate depression, and 75 or more was considered severe depression). The higher the score, the more severe the depression. Cronbach'sα coefficient was 0.874, content validity was 0.853, and internal consistency was good.

(3) Perceived stress scale (PPS) (7): The scale consists of 14 items, and each item uses a 5-level scoring method (0–4). The total score is the sum of the scores of each item, ranging from 0 to 56. The higher the score, the greater the perceived pressure. The Chinese version of PSS has been tested to have sufficient retest reliability (*r* = 0.81), internal consistency (Cronbach's α = 0.85), and structural validity.

### Statistical Analysis

Spss20.0 statistical software was used for data analysis. Descriptive analysis was used for demographic and clinical variables. Measurement data are expressed as mean ± standard deviation, and count data are expressed by frequency or percentage. If two or more sets were measurement data of normal distribution and approximate normal distribution, *t*-test or one-way analysis of variance was used for comparison, while two or more sets of measurement data that do not satisfy the normal distribution were compared using Mann–Whitney *U* nonparametric rank-sum test. Multiple stepwise regression analysis was used to analyze the influencing factors of depression of ICU nurses; *p* < 0.05 indicated statistical significance.

## Results

### General Information

In this survey, the cohort consisted of 14 (17.95%) males and 64 (82.05%) females. The age group ≤ 25-years-old consisted of 13 (16.47%) nurses, 26–39-years-old age group had 62 (79.49%) nurses, and age group ≥40-years-old consisted of 3 (3.85%) nurses. Eighteen (23.08%) nurses had <5 years of experience, 30 (38.46%) nurses had 5–10 years of experience, 17 (21.79%) nurses had 11–15 years of experience, and 13 (16.67%) nurses had >15 years of experience. The level of education was as follows: 18 (23.08%) nurses were at the college level or less, and 60 (76.92%) nurses were undergraduates. Nineteen (24.36%) nurses had the title of general, 36 (46.15%) were senior, and 23 (29.49%) were supervisors and above (Table 1).

**Table 1:** Univariate analysis of depression in ICU nurses combating COVID-19 pandemic (mean ± sd, *n* = 78).

**Project**	**Category**	**Number of** **cases (%)**	**SDS total score** **(points)**	**T/F**	***P*** **-value**	**Project**	**Category**	**Number of cases (%)**	**SDS total score (points)**	**T/F**	***P*** **-value**
Gender	Male	14 (17.95)	45.54 ± 11.004	−2.108	0.038	Infectious ward work experience	Yes	56 (71.79)	53.53 ± 11.512	7.402	0.008
	Female	64 (82.05)	52.64 ± 11.498				No	22 (28.21)	45.85 ± 10.368		
Age (years)	≤ 25	13 (16.67)	50.67 ± 13.680	0.340	0.713	Major epidemic	Yes	39 (50)	52.53 ± 11.7421	0.782	0.397
	26–39	62 (79.49)	51.75 ± 10.931			Experience	No	39 (50)	50.19 ± 11.626		
	≥40	3(3.85)	46.25 ± 20.653			Epidemic prevention control training	yes	74(94.87)	50.22 ± 10.660	0.884	0.397
Working (years) age	<5	18 (23.08)	48.89 ± 10.008	0.514	0.674	Control training	No	4 (5.13)	72.50 ± 10.052		
	5–10	30 (38.46)	52.87 ± 13.491			Knowledge of prevention control	Good	60 (76.92)	50.58 ± 11.127	−1.078	0.285
	11–15	17 (21.79)	52.28 ± 10.498			Work experience of critically ill patients	Average	18 (23.08)	53.96 ± 13.327		
	>15	13 (16.67)	50.10 ± 11.267				Yes	67 (85.9)	49.93 ± 11.469	−2.801	0.006
Education	College above	18 (23.08)	57.99 ± 10.183	8.253	0.005	Work place before COVID-19 pandemic	No	11 (14.1)	60.11 ± 9.256		
	Undergraduate	60 (76.92)	49.38 ± 11.418				Hospital ICU	34 (43.59)	53.20 ± 11.899	3.015	0.055
	Master above	0 (0)					Hospital ward	30 (38.46)	52.42 ± 10.857		
Title	Nurse	19 (24.36)	49.93 ± 11.445	0.187	0.830	Clinical front line working hours	Hospital top three	14 (17.95)	44.64 ± 11.131		
	Senior nurse	36 (46.15)	51.74 ± 12.770				≤ 7 days	13 (16.67)	42.88 ± 10.044	4.461	0.006
	Supervisor	23 (29.49)	51.96 ± 10.368				8–14 days	8 (10.26)	45.78 ± 9.496		
Marital status	Marriage	50 (64.1)	51.75 ± 11.504	3.261	0.044	Pressure perception	15–21 days	8 (10.26)	53.75 ± 7.258		
	Unmarried	27 (34.62)	49.63 ± 11.022				>21 days	49 (62.82)	54.13 ± 11.785		
	Divorce	1 (1.28)	78.75				None	15 (19.23)	43.92 ± 9.553	8.622	0.000
	Yes	27 (34.62)	54.40 ± 12.136	1.692	0.095		Yes slightly larger	33 (42.30)	49.37 ± 9.720		
Only child											
Child	No	51 (65.38)	49.75 ± 11.200				Yes larger afford	28 (35.89)	57.86 ± 11.226		
Sleep condition	Worse	61 (78.21)	53.95 ± 10.847	8.228	0.001	Confident to complete the task	Yes largest crash	2 (2.56)	65.63 ± 7.955		
Condition	Constant	16 (20.51)	42.19 ± 10.119				Yes	69 (88.46)	48.97 ± 9.870	−6.079	0.000
	Get better	1 (1.28)	40.00				No	9 (11.54)	69.72 ± 7.310		
State of health	Good	42 (53.85)	46.55 ± 10.942	15.919	0.000						
	Average	33 (42.31)	55.38 ± 8.652								
	Poor	3 (3.85)	74.58 ± 5.204								

### Depression Status of ICU Nurses Fighting Against COVID-19

According to the SDS scale score, the total depression score of ICU nurses was 51.36 ± 11.667, among which 35 nurses had a score >53, and the prevalence of depression was 44.9%. Among these, 19 (24.4%) were mildly depressed, 13 (16.7%) were moderately depressed, and 3 (3.8%) were severely depressed.

### ICU Nurses' Stress Perceptions Against the COVID-19

The pressure perception of the ICU nurses who fought against the COVID-19 was 33.85 ± 7.749, of which the tension score was 17.41 ± 4.966, and the control sense score was 16.44 ± 5.949. A total of 24 (30.80%) individuals felt normal, 42 (53.80%) felt slightly more stressed, and 12 (15.40%) felt extreme pressure.

### Single-Factor Analysis of ICU Nurses' Depression Status That Affected the COVID-19

The depression status of ICU nurses in different groups was compared by variance analysis. Differences in gender, education, health, sleep, COVID-19 outbreak prevention and control emergency training, front-line working hours, confidence in completing tasks, stress perception assessment and other factors are statistically significant (*p* < 0.05; Table 1).

### Multi-Factor Analysis of ICU Nurses' Depression Status That Affected the COVID-19

Based on the statistically significant factors of difference as the argument, the multi-step regression analysis was carried out with the total depression divided into dependent variables. The total score of stress perception, the total score of critical work experience and the total score of education level enter the regression equation. Among them, critically ill work experience (β = 9.930, *p* < 0.001) has a positive predictive effect on the total score of depression, while the total score of stress perception (β = −0.884, *p* < 0.001) and education (β = −6.061, *p* < 0.001) had a negative predictive effect on the total depression score. The total scores of stress perception, critical work experience, and academic qualifications explained the 52.7% variation in the work input (Table 2).

**Table 2:** Results of multiple stepwise regression of influencing factors of depression.

**Project**	**Partial regression** **coefficient (B)**	**Standard error** **(S.E)**	**Standardization regression** **coefficients**	**t**	***P***
Constant	80.670	5.801		13.906	0.000
Total pressure perception	−0.884	0.122	−0.587	−7.259	0.000
Work experience of critically ill patients	9.930	2.648	0.298	3.751	0.000
Education	−6.061	2.241	−0.220	−2.705	0.000

## Discussion

### Depression Status of ICU Nurses During COVID-19

In this study, the total score of depression was 51.36 ± 11.667. The prevalence of depression between ICU nurses was 44.9% (score of more than 53). This is in line with Nickell et al. Research (8). The prevalence of depression in SARS outbreaks is 45% and may also be affected by related factors of cardiovascular health and interpersonal relationships and familiar violence (9). The depression of ICU nurses is significantly higher than that of the general population, and it also confirms the existence of mental health issues in ICU nurses in China. The working environment of ICU is specific, and severe COVID-19 pandemic patients exhibit complicated conditions and rapid changes. The ICU nurses need to withstand the extremely high risk of infection and often need to give emergency treatment within a few seconds, which is likely to cause tension. Other studies have shown that ICU nurses are high-emotional labor groups, and improper emotional management can aggravate the imbalance of their physical and mental health (10), thereby affecting the quality of nursing work. In the face of such challenges, ICU nurses lack optimism spirit (11), and adequate emotional management can improve the negative emotions of depression of ICU nurses (12). In addition, the use of diversified emotional management, such as system stress management (13), Williams life skills training (14), mindfulness stress reduction therapy (15), reflection (16), etc., can effectively reduce the level of depression of ICU nurses, so that they can actively respond to difficult situations. At present, hospitals rarely carry out emotional management-related training, improve the ICU nurse depression in this regard has a lot of room for improvement.

### Influencing Factors of Depression of ICU Nurses During COVID-19

#### Stress Perception

The COVID-19 pandemic is a stress event and a major cause of depression. Cohen et al. (7) suggest that “perceived stress” is an individual's psychological response to the cognitive evaluation of various stimuli in the environment. Studies have shown that perceived stress positively predicts depression (17). The higher the level of perceived stress, the more severe the depression, which is consistent with studies by Nikcevi et al. (18). ICU nurses are under more pressure than other clinical departments, which are closely related to the ICU's environment and nature of work (19). According to the American Environmental Protection Agency (20), alarm sounds (over 60 dB) are prone to negative emotions such as depression (21). Even at rest, the need to work overtime at all times can lead to tension and difficulty in relaxing. Patients with COVID-19 admitted to the ICU are in critical condition. Critical state as a bad stimulation, easy to cause nurses to produce depression (22), affecting the ICU nurse's internal and external environment, so that the human body into a stress state (23). Studies have shown (24) that social support plays an important role in protecting the physical and mental health of ICU nurses. Therefore, hospital management should provide ICU nurses with adequate logistical support and guidance to express their concerns and concerns, understand their needs, enable them to seek the attention of managers, relieve work stress, and enhance organizational belonging (25). It is recommended that hospitals have full-time psychologists to help ICU nurses relieve psychological stress.

#### Work Experience of Critically Ill Patients

The study showed that the depression score of nurses with critical patient work experience was 49.93 ± 11.469, and that of nurses without critical patient work experience was 60.11 ± 9.256. The difference between the two was statistically significant (*t* = −2.801, *p* = 0.006). Insufficient experience in critical illness may lead to a lack of professional knowledge and unskilled clinical skills, resulting in a psychological burden on nursing COVID-19 patients, leading to job burnout (26). With the increase of nurses' work experience and through systematic training and practice, nurses' professional knowledge and skills have been improved, and they can adapt to the closed and complex ICU environment (27) and reduce depression (28). In order to meet all kinds of difficult and high-level treatment and nursing needs of COVID-19 patients, ICU nurses must possess keen observation ability and comprehensive emergency response ability, including timely detection and active response to changes in COVID-19 patients' conditions, so as to win the golden time of emergency rescue and improve the success rate of emergency rescue (29). Nursing managers should strengthen the standardized training of ICU nurses, constantly cultivate and improve the psychological quality and ability of ICU nurses with less work experience, and constantly generate a sense of accomplishment in practical work, which can effectively reduce the occurrence of depression and other unhealthy mental states (30).

#### Education

In this study, the depression score of undergraduate nurses was 49.38 ± 11.418, and that of undergraduate and below nurses was 57.99 ± 10.183, and the difference was statistically significant. Nurse's education was negatively correlated with depression, and the lower the nurse's education, the more severe the depression, contrary to the findings of Prasetyo et al. (31). The lack of a comprehensive analysis of the condition and problems of coVID-19 critically ill patients at a low level of education may reduce their professional values and professional identity and lead to depression (32). ICU nurses with bachelor's degree or above have better comprehensive quality and thinking ability than those with junior college degree or below. They have systematic learning ability of ICU specialized knowledge and skills, strong understanding and thinking ability, and have strong basic skills to deal with work difficulties. These factors make them actively adopt COVID-19 knowledge. In the treatment of critically ill patients with COVID-19, positive attitude and high stress resistance had little effect on mental health (33). The critical factor is the emergency deployment of human resources to support the ICU. Psychologists should be invited to provide psychological counseling for ICU nurses with low academic qualifications to improve their mental health status (34). At the same time, stratified training and education should be provided for nurses at all levels to improve their professional skills (35), provide opportunities and platforms for personal development, enhance nurses' sense of self-worth and professionalism, and improve their working conditions (36).

In conclusion, during the COVID-19 pandemic, stress perception score, work experience in critical care and education background were important factors affecting the occurrence of depression in ICU nurses. Medical institutions should timely understand the depression of ICU nurses and carry out targeted psychological interventions to avoid severe post-traumatic stress disorder in the future (37). Limitations of this study: First, since “Questionnaire Star” is not a field survey, respondents can freely fill in the questionnaire without signature, so it is impossible to check and evaluate the authenticity of the information obtained. Second, the sample size of the study is insufficient, which does not involve multi-center research and has limited representativeness. In the future, the scope of the study can be expanded to formulate intervention models in line with professional characteristics.

## Data Availability Statement

The original contributions presented in the study are included in the article/supplementary material, further inquiries can be directed to the corresponding author/s.

## Ethics Statement

The studies involving human participants were reviewed and approved by the Ethics Committee of Beijing Ditan Hospital Capital Medical University (2020–023–01). Written informed consent from the participants was not required to participate in this study in accordance with the national legislation and the institutional requirements.

## Author Contributions

JL and YZ: substantially contributed to conception or design. YH: contributed to acquisition, analysis, or interpretation of data. JL and YB: drafted the manuscript for important content. LL and WY: critically revised the manuscript for important intellectual content. All authors gave final approval of the manuscript.

## Conflict of Interest

The authors declare that the research was conducted in the absence of any commercial or financial relationships that could be construed as a potential conflict of interest.

## Publisher's Note

All claims expressed in this article are solely those of the authors and do not necessarily represent those of their affiliated organizations, or those of the publisher, the editors and the reviewers. Any product that may be evaluated in this article, or claim that may be made by its manufacturer, is not guaranteed or endorsed by the publisher.
